# Genome-Wide Association Study of Meat Quality Traits in Nellore Cattle

**DOI:** 10.1371/journal.pone.0157845

**Published:** 2016-06-30

**Authors:** Ana F. B. Magalhães, Gregório M. F. de Camargo, Gerardo A. Fernandes, Daniel G. M. Gordo, Rafael L. Tonussi, Raphael B. Costa, Rafael Espigolan, Rafael M. de O. Silva, Tiago Bresolin, Willian B. F. de Andrade, Luciana Takada, Fabieli L. B. Feitosa, Fernando Baldi, Roberto Carvalheiro, Luis A. L. Chardulo, Lucia G. de Albuquerque

**Affiliations:** 1 Departamento de Zootecnia, Faculdade de Ciências Agrarias e Veterinárias, Jaboticabal, São Paulo, Brazil; 2 Conselho Nacional de Desenvolvimento Científico e Tecnológico – CNPq, Brasília, Distrito Federal, Brazil; 3 Departamento de Melhoramento e Nutrição Animal, Faculdade de Medicina Veterinária e Zootecnia, Botucatu, São Paulo, Brazil; University of Bonn, GERMANY

## Abstract

The objective of this study was to identify genomic regions that are associated with meat quality traits in the Nellore breed. Nellore steers were finished in feedlots and slaughtered at a commercial slaughterhouse. This analysis included 1,822 phenotypic records of tenderness and 1,873 marbling records. After quality control, 1,630 animals genotyped for tenderness, 1,633 animals genotyped for marbling, and 369,722 SNPs remained. The results are reported as the proportion of variance explained by windows of 150 adjacent SNPs. Only windows with largest effects were considered. The genomic regions were located on chromosomes 5, 15, 16 and 25 for marbling and on chromosomes 5, 7, 10, 14 and 21 for tenderness. These windows explained 3,89% and 3,80% of the additive genetic variance for marbling and tenderness, respectively. The genes associated with the traits are related to growth, muscle development and lipid metabolism. The study of these genes in Nellore cattle is the first step in the identification of causal mutations that will contribute to the genetic evaluation of the breed.

## Introduction

Brazil is one of the world’s largest exporter of beef and possesses the second largest herd [1]. Zebu animals (*Bos taurus indicus*) account for 80% of this herd and the Nellore breed corresponds to 90% of these animals [2]. Studies have shown that the quality of *Bos taurus indicus* meat is inferior when compared to the meat of *Bos taurus taurus* [3,4]. Meat quality is a generic term used to describe consumer perceptions of meat [5]. In this respect, tenderness is one of the most important attributes that determines the quality of the product and consumer acceptance [6]. Another attribute, defined by sensory panel testing, which is involved in the appreciation of meat quality, is intramuscular fat [7]. This attribute affects the flavor, juiciness and chewing of meat. These traits are important to guarantee the stability and market expansion of Brazil as a beef exporter.

The application of traditional selection to these traits is difficult, since they are expensive and difficult to measure because it requires the slaughter of the animals. Genome-wide association studies (GWAS) allow the identification of single-nucleotide polymorphisms (SNPs) associated with large-effect genes that influence these traits, providing a better biological understanding of the trait and a list of candidate genes for fine mapping.

At first, classical methods were used for GWAS, which were based on testing one marker at a time. However, using those methods increases the chance of false-positives due to multicolinearity [8]. Furthermore, it is difficulty to test the significance of each marker considering the existence of thousands of SNPs [9]. Currently, different methods of modeling SNPs simultaneously have been used. In the majority of these methods, the analyses are carried out in multiple steps, which first require traditional genetic evaluation using an animal model, followed by the formation of pseudo-phenotypes and, finally, the estimation of the SNP effects [10]. In addition, those methods generally require that phenotype and genotype information are available for all animals. Recently, a method that uses pedigree, phenotype and genotype data in a single step (ssGBLUP) was proposed [11]. This method can be easily implemented and allows including in the analysis more animals with phenotypes than genotypes.

Some association studies of SNPs with meat quality traits in taurine cattle have identified different regions associated with marbling, which are located on chromosomes 2, 3, 7, 8, 10, 17, 22 and 27 and with meat tenderness, which are located on chromosomes 7, 8, 9, 10 and 29 [12–15]. Genes that affect meat tenderness, such as calpain (CAPN1) and calpastatin (CAST), are located on chromosomes 29 and 7, respectively [16,17]. The SNPs CAPN4751, CAPN4753, UOGCAST and WSUCAST, which are associated with tenderness in *Bos taurus taurus*, are also polymorphic in Nellore cattle [18]. In GWAS of Nellore animals, [19] identified SNPs for tenderness located near CAPN1, CAPN2, CAPN5 and CAST.

In previous studies, it is unclear whether these genes contribute to the additive genetic variance of these traits in Zebu cattle. Therefore, the objective of this study was to perform GWAS in order to identify genomic regions that are associated with meat quality traits in the Nellore breed.

## Materials and Methods

### Data

Tenderness and marbling were the meat quality traits used in this study. Data of male Nellore animals born between 2008 and 2010 were collected among herds located in different regions of Brazil, which belong to three breeding programs (DeltaGen, Paint, and Nelore Qualitas). The animals were finished in feedlots for approximately 90 days and slaughtered at an average age of 731 ± 81 days.

The approval of the ethics committee of the Faculty of Agrarian Sciences and Veterinary of Sao Paulo State (FCAV-UNESP) was not necessary, because the slaughter of animals was done in commercial slaughterhouses (JBS S/A, Minerva S.A and Marfrig Alimentos S.A), located in several regions of Brazil. Since such slaughterhouses have animal welfare department, staffed by professionals trained by WAG (World Animal Protection), ensuring that the animals are killed humanely, by use of captive bolt pistol in the stunning process.

The carcasses were maintained in a cold chamber for 24 to 48 h *post-mortem* and *longissimus dorsi* muscle (tenderloin) samples were detached with bone between the 12^th^ and 13^th^ rib of the left half-carcasses. All samples were frozen and none of them was aged. Animals from the same farm and year of birth were slaughtered in the same slaughterhouse and slaughter date.

*Longissimus dorsi* samples measuring 2.54 cm in thickness were obtained for analysis of tenderness. The procedure standardized and proposed by [20] was adopted, which consists of the measurement of shear force using a mechanical Salter Warner-Bratzler Shear Force device. The degree of marbling was scored on a scale from 1 to 10 according to the method of [21], where 1 = practically absent; 2 = traces; 3 = slight; 4 = small; 5 = modest; 6 = moderate; 7 = slightly abundant; 8 = moderately abundant; 9 = abundant, and 10 = very abundant.

There were a total of 7,436 animals in the relationship matrix. Data from 1,875 animals with tenderness and marbling records were available. Measures that were 3.5 standard deviations above or below the mean of the contemporary group (year, farm and management group at yearling) were excluded from the analyses. Additionally, contemporary groups containing fewer than three animals were eliminated. Thus, there were records of 1,822 animals with tenderness measures and of 1,873 animals with marbling measures (Table 1). The heritabilities were estimated using Bayesian approach with BLUPF90 family of programs [22]. The model included fixed effects of contemporary groups (year, farm of birth and management group at yearling) and age at slaughter and days between slaughter and physicochemical analysis of meat as covariates (linear effect).

**Table 1:** Descriptive statistics and heritability (h^2^) estimates of meat quality traits in Nellore cattle.

Traits	N	Mean	SD	Med	h^2^	SE
Tenderness (kg)	1,822	5.17	1.37	5.04	0.12	0.07
Marbling	1,873	-	-	2.80	0.10	0.07

N: number of observations; SD: standard deviation; Med: Median; h^2^: heritability; SE: Standard error

The animals were genotyped using a high-density panel containing 777,962 SNPs (Illumina High-Density Bovine BeadChip). The criteria for exclusion of SNPs were: non-autosomal SNPs, SNPs at the same position, with a minor allele frequency ≤ 0.02, a p value for Hardy-Weinberg equilibrium ≤ 10^−5^, GenCall score ≤ 0.70, call rate ≤ 0.98, and r² (correlation between SNPs) > 0.995 with adjacent SNPs within a window of 100 SNPs. Samples with a call rate ≤ 0.92 and without a valid phenotype were also excluded. After quality control, 1,630 animals genotyped for tenderness, 1,633 genotyped for marbling, and 369,722 SNPs remained.

### GWAS

The ssGWAS method proposed by [9] was used for GWAS. A single-trait model was considered for the studied traits:

y=Xb+Za+e

where *y* is the vector of phenotypic observations; *X* is an incidence matrix relating the phenotypes to the fixed effects; *b* is the vector of fixed effects, including the contemporary group (year, farm and management group at yearling) and slaughter age and days between slaughter and physicochemical analysis of meat as covariates (linear effect); *Z* is an incidence matrix relating the animal to the phenotype; *a* is the vector of effects of the animals, and *e* is the vector of residual effects.

The variances of *a* and *e* can be written as:

$$Var\left[\begin{array}{c} a\\ e \end{array}\right]=\left[\begin{array}{cc} H\sigma_{a}^{2} & 0\\ 0 & I\sigma_{e}^{2} \end{array}\right]$$

where *σ*^*2*^_a_ is the direct additive genetic variance; *σ*^*2*^_e_ is the residual variance; *H* is a matrix that combines pedigree and genomic information as proposed by [10], and *I* is an identity matrix. The inverse of matrix *H* is:

$$H^{-1}=A^{-1}+\left[\begin{array}{cc} 0 & 0\\ 0 & G^{-1}-A_{22}^{-1} \end{array}\right]$$

where *A* is the pedigree matrix for all animals; *A*_22_ is the relationship matrix for genotyped animals, and *G* is the genomic relationship matrix, which was calculated as described by [23].

The effects of the SNPs (ȗ) were obtained using the equation described by [9]:

$$\overset{⌢}{u}={\lambda \text{DZ}}^{\text{'}}G^{*-1}{\overset{⌢}{a}}_{g}$$

where ȗ is the vector of SNP effects; λ is the variance ratio calculated according to [23]; ȃ_g_ is the animal effect of genotyped animals; Z is a matrix that relates the genotypes of each locus; D is a diagonal matrix of the weights of SNP variances. For this study the weights of SNP was not used, so D = I (identity matrix). G is the genomic relationship matrix.

The analyses were carried out using the BLUPF90 family of programs [22]. The results of GWAS are reported as the proportion of variance explained by a window of 150 adjacent SNPs. The MapViewer tool of the bovine genome, available at NCBI (http://www.ncbi.nlm.nih.gov/projects/mapview/map_search.cgi?taxid=9913&build=103.1), was used to identify the genes. The DAVID software [24] was used to describe the genes.

## Results and Discussion

**Fig 1:**
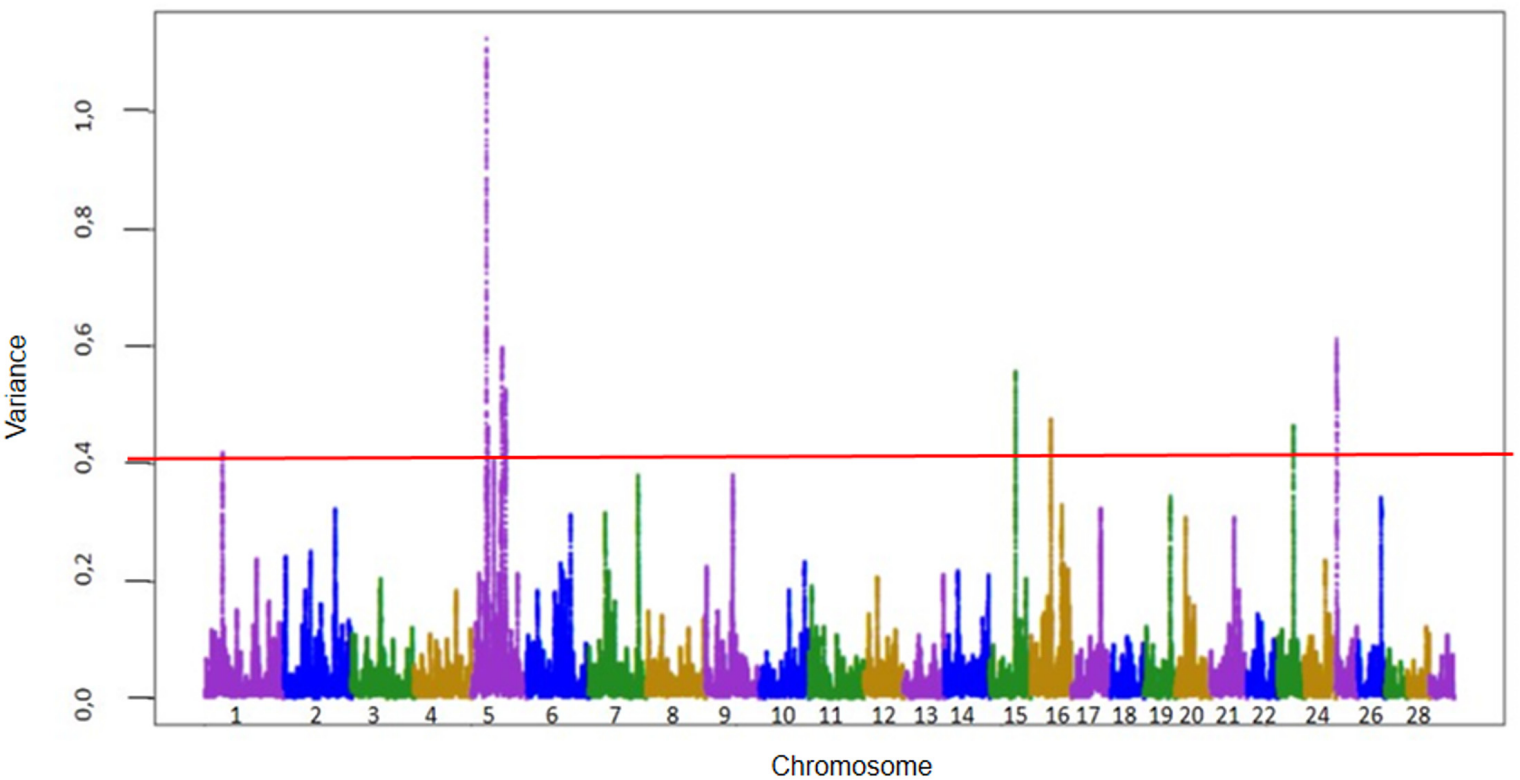
Manhattan plot of additive genetic variance explained by windows of 150 adjacent SNPs for Marbling.

**Fig 2:**
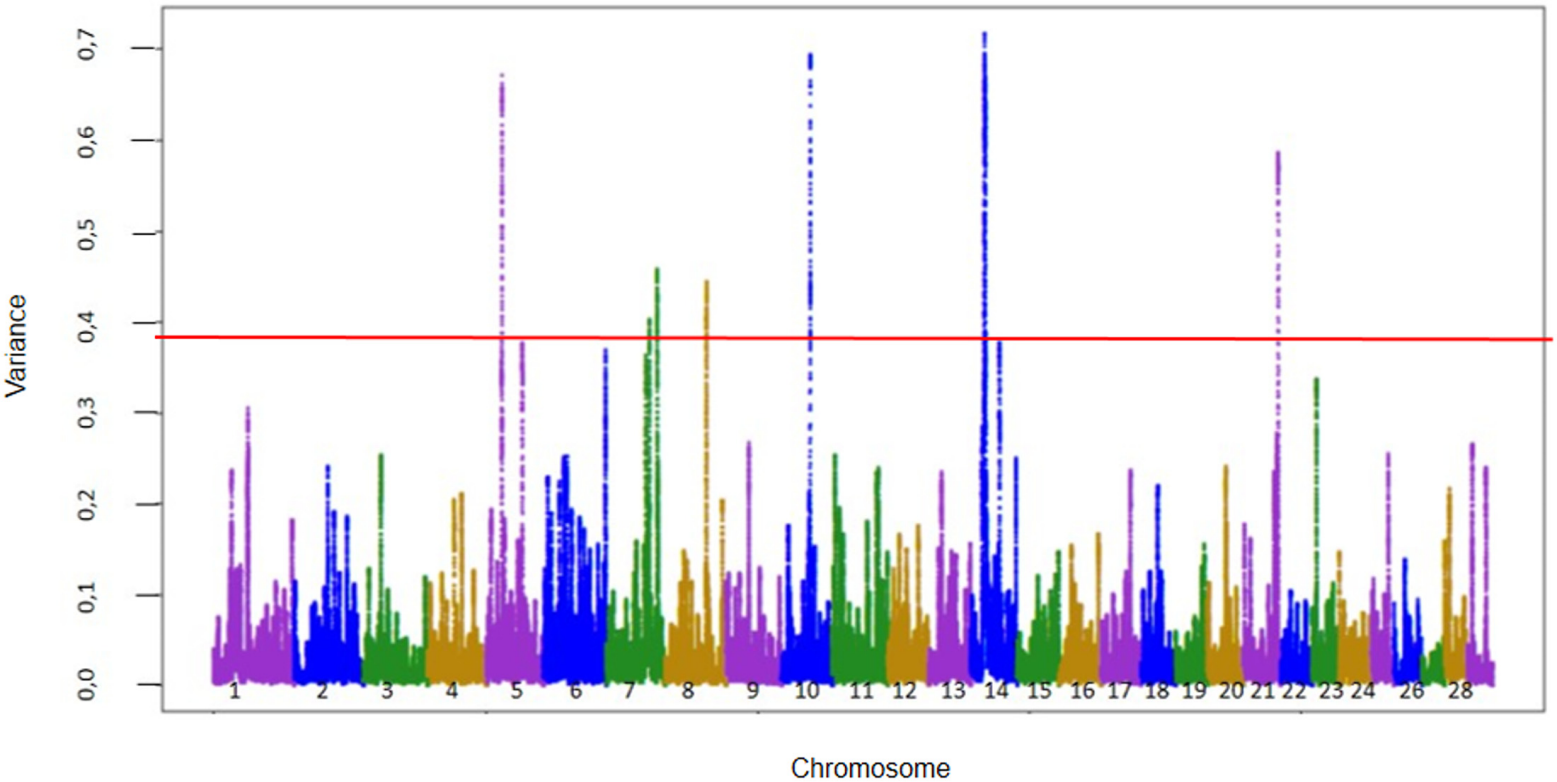
Manhattan plot of additive genetic variance explained by windows of 150 adjacent SNPs for Tenderness.

Figs 1 and 2 show the Manhattan plot with the percentages of additive genetic variance explained by windows of 150 adjacent SNPs for marbling and tenderness, respectively. Only genes inside windows with the highest percentage of additive genetic variance were presented. Others windows to complete top 10 ranking windows for genetic variance, as described by [25] are in S1 and S2 Tables and shows the chromosome, location, identification of genes and proportion of genetic variance for traits of marbling and tenderness. General information about all results of ssGWAS for both traits are in S1 File.

The windows are located on chromosomes 5, 15, 16 and 25 for marbling and on chromosomes 5, 7, 10, 14 and 21 for tenderness and explained 3.89% and 3.80% of the additive genetic variance for marbling and tenderness, respectively.

Describing the results obtained from the genes for marbling and tenderness (Tables 2 and 3), it was observed that these traits have a similar window (31349253bp– 32177734bp) in the chromosome 5. For marbling, this window is the one that explains the highest percentage of additive genetic variance (1.12%) and for tenderness it is the third largest effect window (0.67%). This is an interesting result to be analyzed since the traits are moderately correlated [26,27]. Therewith, it is possible identify SNPs associated the candidate genes that exert a pleiotropic effect on meat quality traits and contributes to the genetic evaluation of both traits. Using the DAVID software, ten genes that act as olfactory receptors of the same window were identified *(LOC617415*, *LOC507383*, *LOC504567*, *LOC523258*, *LOC509817*, *LOC781968*, *LOC515887*, *LOC506992*, *LOC617388*, *LOC781446)*. This cluster of genes participates in the change of GDP (guanosine diphosphate) to GTP (guanosine triphosphate), which are regulators of G proteins. GDP and GTP can be used as energy source and are responsible for transferring energy within the cell [28]. Another explanation for the effect of olfactory receptors on meat traits is their action in the duodenum, where they promote the absorption of fatty acids and thus increase the accumulation of fat as demonstrated in mice [29]. Furthermore, olfactory receptors are known to act on adipose tissue and adipocyte differentiation, increasing fat accumulation [30]. Some studies also suggest that olfactory receptors are involved in food intake because of their known action on olfactory neurons, increasing the search for food and food intake [31], and consequently weight gain and fat accumulation. Analysis of copy number variations for polyunsaturated fatty acids in the same databank also identified olfactory receptors [32]. This shows the strong influence of this group of genes on meat traits in Nellore cattle. Genes play multiple roles in metabolism. In this respect, olfactory receptors were first described to be involved in the perception of odors, but it cannot be ruled out that they exert other functions in the organism as mentioned above. Thus, specific studies demonstrating the true role of this group of genes in lipid metabolism are needed.

**Table 2:** Chromosome (Chr), location, identification of genes and proportion of variance (VAR) explained by windows with largest effects on marbling^a^.

Chr	Location (bp)	Genes	VAR (%)
5	31349253–32177734	LALBA, LOC104972406, LOC784038	1.12409
		LOC526630, LOC614370, LOC515887	
		LOC506992, LOC781446, LOC509508	
		LOC781517, LOC507383, LOC781595	
		OR8S1, LOC504567, LOC781756, ZNF641	
		LOC781840, LOC101907855, LOC617415,	
		C5H12orf54, LOC509817, LOC781968, H1FNT	
		LOC617388, LOC617380, LOC518129	
		LOC100300010, LOC523258, LOC100848234	
25	2252680–2891421	PRSS33, TCEB2, FLYWCH2, NAA60, CASP16	0.61217
		FLYWCH1, KREMEN2, PAQR4, ZNF200	
		PKMYT1, CLDN9, CLDN6, ZNF597, TIGD7	
		TNFRSF12A, HCFC1R1, THOC6, ZNF174	
		CCDC64B, MMP25, IL32, MEFV, ZNF75A	
		LOC100139916, LOC100141258, ZNF263,	
		ZSCAN10, ZNF205, ZNF213, C25H16orf90	
5	68620356–69478777	CHST11, LOC101903254, LOC104972484	0.59773
		SLC41A2, C5H12orf45, ALDH1L2, APPL2,	
		KIAA1033, C5H12orf75, LOC104972485	
15	54775627–55624618	CHRDL2, LOC100139754, RNF169, GDPD5	0.55671
		LOC100848131, XRRA1, NEU3, KLHL35	
		LOC101904814, LOC507022, OR2AT4	
		SPCS2, LOC512627, LOC790343, MIR326	
		SLCO2B1, TPBGL, ARRB1, MAP6, RPS3	
		LOC104974272, SERPINH1, LOC101905131	
5	76214455–77325534	ELFN2, MFNG, CARD10, USP18, ALG10, SYT10	0.52581
16	43778493–44690610	CASZ1, LOC101906968, PEX14, TRNAG-CCC	0.47581
		DFFA, CORT, APITD1, PGD, CLSTN1, RBP7	
		LOC101904547, TRNAR-CCG, CTNNBIP1	
		KIF1B, LOC101904590, LOC104974439, PIK3CD	
		LOC104974446, UBE4B, LOC101905440	
		LOC104974441, LZIC, LOC104974440, NMNAT1	

^a^NCBI Symbol (Assembly UMD3.1, annotation release 103).

**Table 3:** Chromosome (Chr), location, identification of genes and proportion of variance (VAR) explained by windows with largest effects on tenderness^a^.

Chr	Location (bp)	Genes	VAR (%)
14	23048646–23659905	ST18, LOC101906592, FAM150A, LYPLA1	0.71742
		RB1CC1, LOC104974017, TCEA1	
		NPBWR1, OPRK1, ATP6V1H, RGS20	
10	59864007–60992988	TRPM7, LOC101906880, USP50, SLC27A2	0.69363
		USP8, LOC101906954, GABPB1, ATP8B4	
		HDC, LOC100848857, LOC104973174	
		LOC101907420, DTWD1, FAM227B	
5	31349253–32177734	LALBA, LOC104972406 H1FNT, LOC617415	0.67148
		LOC526630, LOC614370, LOC515887	
		LOC506992, LOC781446, LOC509508	
		LOC781517, LOC507383, LOC781595	
		OR8S1, LOC504567, LOC781756, LOC100848234, ZNF641	
		LOC781840, LOC101907855, LOC784038	
		C5H12orf54, LOC509817, LOC781968	
		LOC617388, LOC617380, LOC518129	
		LOC100300010, LOC523258	
14	24336953–25030555	XKR4, TRNAT-AGU, TMEM68, LOC104974019	0.66924
		TGS1, LYN, RPS20, MOS, PLAG1	
21	65833836–66414612	BCL11B, SETD3, CCNK, HHIPL1, EML1	0.58616
		CCDC85C, LOC101906687, CYP46A1	
7	98394404–98767591	CAST, LOC104968992, ERAP1	0.45783

^a^NCBI Symbol (Assembly UMD3.1, annotation release 103).

For marbling, the genes described in Table 2 are related to carbohydrate and lipid metabolism. This fact agrees with the physiology of interspersed fat deposition in muscle, since lipids are derived from the consumption of fats or from the excess intake of carbohydrates that are processed and stored in the form of fat. The *TCEB2* gene was detected by proteomic analysis of hepatic fat droplets, which showed that high production of this protein protects against diabetes in mice [33]. The *TIGD7* gene has been associated with body mass index and plasma glucose levels in humans [34]. The *APPL2* gene has been shown to be associated with obesity in humans [35] and to play a role in the inhibition of glucose uptake in skeletal muscle [36]. The *NEU3* gene promotes the accumulation of triglycerides in mice, which may facilitate the deposition of body fat [37]. The *MFNG* gene is responsible for causing bile duct abnormalities in mice, which can compromise bile flow and the consequent emulsification of fat for absorption [38]. The list of genes for marbling was compiled using the DAVID software. The *CHST11*, *LALBA* and *PGD* genes form a cluster and are involved in the chemical reactions resulting in the formation of carbohydrates.

The genes associated with marbling in the present study are not the same as those reported in other GWAS for the same trait in *Bos taurus taurus* [39–41]. In study with the same breed, [42], the authors also reported that the regions that most influenced the trait did not coincide with those of the present study.

For meat tenderness, genes related to growth and muscle development were identified (*PLAG1*, *SLC27A2*, *RB1CC1*, *HDC*, *LYPLA1*, *XKR4* and *TMEM68**)*. The PLAG1 gene has been associated with meat tenderness in cattle [43]. This gene also is associated with carcass traits in a study on the same animals [44], in addition to other studies, with different breeds [39,45–48]. This gene has also been associated with growth traits, feed intake and reproductive and andrological traits in different breeds of the same species [39,43,49–54]. This gene has an important pleiotropic effect and can be an excellent candidate for a large-effect gene because it acts on different traits. This fact can be explained by the function of the gene, which is a transcription factor for the growth hormone IGF-2 [55].

The *SLC27A2* gene has also been associated with meat tenderness in cattle [56], among other carcass traits [57]. The *RB1CC1* gene is involved in muscle development and possesses non-synonymous SNPs that are associated with pectoral muscle size in broiler chickens [58]. Furthermore, the gene acts on skeletal muscle of chicken under thermal stress [59]. Since this gene acts through temperature variations in the living animal, its activity may continue postmortem. A decline in temperature is known to affect meat tenderness. Thus, different variants of the gene may act in the carcass through postmortem temperature variation, resulting in more or less tender meat.

The *HDC* gene is also expressed in skeletal muscle where it is involved in the processing of histamine, but its function is unknown [60]. The *SETD3* gene participates in the differentiation of muscle cells [61].

The *LYPLA1*, *XKR4* and *TMEM68* genes have been associated with feed intake and growth in cattle [62]. The *XKR4* gene has been associated with fat thickness in cattle [41,63] and the *LYN* and *TGS1* genes with carcass phenotypes also in cattle [41]. Furthermore, in another study with same population as in this one [64], *XKR4*, *TMEM68*, *TGS1* and *LYN* genes were associated to birth weight. All genes mentioned above, are located in chromosome 14 and were associated with several traits besides tenderness, suggesting a pleiotropic effect. These results corroborate those describing positive genetic correlation estimates of tenderness with growth traits [65] and with fat thickness [66].

The calpastatin (*CAST*) is an important gene associated with tenderness, which is known to inhibit calpain (*CAPN1*). These genes (*CAST* and *CAPN1*) are considered responsible for the process of meat tenderization during the postmortem period [67]. *CAPN1* determines an increase in final meat tenderness, while *CAST* acts in the opposite manner, inhibiting this tenderizing process. In the present study, the window where *CAST* was located explained 0.46% of the additive genetic variance in tenderness. The *CAST* gene has been associated with meat tenderness in GWAS of Australian cattle [68]. However, studying approximately 500 Nellore animals, [19] found no association between the *CAST* gene and tenderness. The authors identified 56 genes for different tenderness measures (24 h, 7 and 14 days after slaughter), but these genes only explained a small percentage of the additive genetic variance (less than 0.2%).

Different genes have been reported in other studies conducted on the same breed and traits [19,42], indicating the existence of a genetic difference between populations analyzed within breeds. Several factors may be responsible for these differences, such as variations in allele frequency, linkage disequilibrium, coverage of the SNP chip in the breed, method and number of animals, since the studies on Nellore animals cited above used few animals when compared to the present study.

Important genomic regions associated with meat quality traits were identified in the present study, providing a better biological understanding of tenderness and marbling in *Bos taurus indicus*. The identification of these genes in cattle using genomic tools is the first step in the search for causal mutations that exert great influence on traits of economic interest and can contribute in the future to genetic evaluations.

## Supporting Information

S1 File Results of ssGWAS for marbling and tenderness. (XLS)

S1 Table Chromosome (Chr), location, identification of genes and proportion of variance (VAR) explained by windows with largest effects on marbling^a^. ^a^NCBI Symbol (Assembly UMD3.1, annotation release 103).
(DOCX)

S2 Table Chromosome (Chr), location, identification of genes and proportion of variance (VAR) explained by windows with largest effects on tenderness^a^. ^a^NCBI Symbol (Assembly UMD3.1, annotation release 103).
(DOCX)
