# Supplementary material for: Genome-Wide Association Study of Meat Quality Traits in Nellore Cattle
Source: PLoS One. 2016 Jun 30;11(6):e0157845. doi: 10.1371/journal.pone.0157845 (PMC4928802; doi:10.1371/journal.pone.0157845)
Supplement: S1 Table — aNCBI Symbol (Assembly UMD3.1, annotation release 103). (DOCX) [file pone.0157845.s002.docx]

| Chr | Location (bp) | Genes | VAR (%) |
| --- | --- | --- | --- |
| 23 | 30597826-31290379 | [LOC528373](http://www.ncbi.nlm.nih.gov/entrez/query.fcgi?db=gene&cmd=retrieve&dopt=full_report&list_uids=528373), [OR2B6](http://www.ncbi.nlm.nih.gov/entrez/query.fcgi?db=gene&cmd=retrieve&dopt=full_report&list_uids=528374), [LOC783002](http://www.ncbi.nlm.nih.gov/entrez/query.fcgi?db=gene&cmd=retrieve&dopt=full_report&list_uids=783002), [HIST1H3C](http://www.ncbi.nlm.nih.gov/entrez/query.fcgi?db=gene&cmd=retrieve&dopt=full_report&list_uids=616800) | 0.46498 |
|  |  | [HIST1H2BI](http://www.ncbi.nlm.nih.gov/entrez/query.fcgi?db=gene&cmd=retrieve&dopt=full_report&list_uids=616776), [HIST1H1B](http://www.ncbi.nlm.nih.gov/entrez/query.fcgi?db=gene&cmd=retrieve&dopt=full_report&list_uids=527304), [LOC529277](http://www.ncbi.nlm.nih.gov/entrez/query.fcgi?db=gene&cmd=retrieve&dopt=full_report&list_uids=529277), [HIST1H2BB](http://www.ncbi.nlm.nih.gov/entrez/query.fcgi?db=gene&cmd=retrieve&dopt=full_report&list_uids=614958) |  |
|  |  | [LOC528329](http://www.ncbi.nlm.nih.gov/entrez/query.fcgi?db=gene&cmd=retrieve&dopt=full_report&list_uids=528329), [LOC527645](http://www.ncbi.nlm.nih.gov/entrez/query.fcgi?db=gene&cmd=retrieve&dopt=full_report&list_uids=527645), [HIST1H2AG](http://www.ncbi.nlm.nih.gov/entrez/query.fcgi?db=gene&cmd=retrieve&dopt=full_report&list_uids=616790), [LOC616868](http://www.ncbi.nlm.nih.gov/entrez/query.fcgi?db=gene&cmd=retrieve&dopt=full_report&list_uids=616868) |  |
|  |  | [LOC100295221](http://www.ncbi.nlm.nih.gov/entrez/query.fcgi?db=gene&cmd=retrieve&dopt=full_report&list_uids=100295221), [LOC616819](http://www.ncbi.nlm.nih.gov/entrez/query.fcgi?db=gene&cmd=retrieve&dopt=full_report&list_uids=616819), [HIST1H2AJ](http://www.ncbi.nlm.nih.gov/entrez/query.fcgi?db=gene&cmd=retrieve&dopt=full_report&list_uids=783767) [MIR2379](http://www.ncbi.nlm.nih.gov/entrez/query.fcgi?db=gene&cmd=retrieve&dopt=full_report&list_uids=100313177) [ZNF322](http://www.ncbi.nlm.nih.gov/entrez/query.fcgi?db=gene&cmd=retrieve&dopt=full_report&list_uids=783152), [ABT1](http://www.ncbi.nlm.nih.gov/entrez/query.fcgi?db=gene&cmd=retrieve&dopt=full_report&list_uids=539270),[LOC521167](http://www.ncbi.nlm.nih.gov/entrez/query.fcgi?db=gene&cmd=retrieve&dopt=full_report&list_uids=521167), [LOC514235](http://www.ncbi.nlm.nih.gov/entrez/query.fcgi?db=gene&cmd=retrieve&dopt=full_report&list_uids=514235) |  |
|  |  | [LOC101906102](http://www.ncbi.nlm.nih.gov/entrez/query.fcgi?db=gene&cmd=retrieve&dopt=full_report&list_uids=101906102), [MIR7857](http://www.ncbi.nlm.nih.gov/entrez/query.fcgi?db=gene&cmd=retrieve&dopt=full_report&list_uids=102466867), [ZNF184](http://www.ncbi.nlm.nih.gov/entrez/query.fcgi?db=gene&cmd=retrieve&dopt=full_report&list_uids=515674), [ZNF391](http://www.ncbi.nlm.nih.gov/entrez/query.fcgi?db=gene&cmd=retrieve&dopt=full_report&list_uids=513014) |  |
|  |  | [POM121L2](http://www.ncbi.nlm.nih.gov/entrez/query.fcgi?db=gene&cmd=retrieve&dopt=full_report&list_uids=100140439), [PRSS16](http://www.ncbi.nlm.nih.gov/entrez/query.fcgi?db=gene&cmd=retrieve&dopt=full_report&list_uids=614489), [HIST1H2AH](http://www.ncbi.nlm.nih.gov/entrez/query.fcgi?db=gene&cmd=retrieve&dopt=full_report&list_uids=616634), [HIST1H2BN](http://www.ncbi.nlm.nih.gov/entrez/query.fcgi?db=gene&cmd=retrieve&dopt=full_report&list_uids=616627) |  |
|  |  | [HIST1H2BJ](http://www.ncbi.nlm.nih.gov/entrez/query.fcgi?db=gene&cmd=retrieve&dopt=full_report&list_uids=522960), [LOC515414](http://www.ncbi.nlm.nih.gov/entrez/query.fcgi?db=gene&cmd=retrieve&dopt=full_report&list_uids=515414) |  |
| 5 | 34192005-35234222 | [SLC38A1](http://www.ncbi.nlm.nih.gov/entrez/query.fcgi?db=gene&cmd=retrieve&dopt=full_report&list_uids=527491), [SCAF11](http://www.ncbi.nlm.nih.gov/entrez/query.fcgi?db=gene&cmd=retrieve&dopt=full_report&list_uids=538632), [TRNAC-ACA](http://www.ncbi.nlm.nih.gov/entrez/query.fcgi?db=gene&cmd=retrieve&dopt=full_report&list_uids=100170901), ARID2[LOC104972414](http://www.ncbi.nlm.nih.gov/entrez/query.fcgi?db=gene&cmd=retrieve&dopt=full_report&list_uids=104972414), [ANO6](http://www.ncbi.nlm.nih.gov/entrez/query.fcgi?db=gene&cmd=retrieve&dopt=full_report&list_uids=521784), [LOC784769](http://www.ncbi.nlm.nih.gov/entrez/query.fcgi?db=gene&cmd=retrieve&dopt=full_report&list_uids=784769) | 0.46182 |
| 1 | 35016832-35918033 | [POU1F1](http://www.ncbi.nlm.nih.gov/entrez/query.fcgi?db=gene&cmd=retrieve&dopt=full_report&list_uids=282315), [LOC104970650](http://www.ncbi.nlm.nih.gov/entrez/query.fcgi?db=gene&cmd=retrieve&dopt=full_report&list_uids=104970650), [HTR1F](http://www.ncbi.nlm.nih.gov/entrez/query.fcgi?db=gene&cmd=retrieve&dopt=full_report&list_uids=407196), [LOC104970808](http://www.ncbi.nlm.nih.gov/entrez/query.fcgi?db=gene&cmd=retrieve&dopt=full_report&list_uids=104970808) | 0.41792 |
|  |  | [CGGBP1](http://www.ncbi.nlm.nih.gov/entrez/query.fcgi?db=gene&cmd=retrieve&dopt=full_report&list_uids=613824), [LOC100335242](http://www.ncbi.nlm.nih.gov/entrez/query.fcgi?db=gene&cmd=retrieve&dopt=full_report&list_uids=100335242), [LOC104970809](http://www.ncbi.nlm.nih.gov/entrez/query.fcgi?db=gene&cmd=retrieve&dopt=full_report&list_uids=104970809) |  |
|  |  | [C1H3orf38](http://www.ncbi.nlm.nih.gov/entrez/query.fcgi?db=gene&cmd=retrieve&dopt=full_report&list_uids=511707), [LOC101904492](http://www.ncbi.nlm.nih.gov/entrez/query.fcgi?db=gene&cmd=retrieve&dopt=full_report&list_uids=101904492) |  |
| 5 | 47069837-47926421 | [LOC100337478](http://www.ncbi.nlm.nih.gov/entrez/query.fcgi?db=gene&cmd=retrieve&dopt=full_report&list_uids=100337478), [TRNAC-GCA](http://www.ncbi.nlm.nih.gov/entrez/query.fcgi?db=gene&cmd=retrieve&dopt=full_report&list_uids=100170902), [LOC525592](http://www.ncbi.nlm.nih.gov/entrez/query.fcgi?db=gene&cmd=retrieve&dopt=full_report&list_uids=525592) | 0.40808 |
|  |  | LOC104969784, [HELB](http://www.ncbi.nlm.nih.gov/entrez/query.fcgi?db=gene&cmd=retrieve&dopt=full_report&list_uids=614572), [LOC783726](http://www.ncbi.nlm.nih.gov/entrez/query.fcgi?db=gene&cmd=retrieve&dopt=full_report&list_uids=783726), [TRNAK-CUU](http://www.ncbi.nlm.nih.gov/entrez/query.fcgi?db=gene&cmd=retrieve&dopt=full_report&list_uids=100170896)[IRAK3](http://www.ncbi.nlm.nih.gov/entrez/query.fcgi?db=gene&cmd=retrieve&dopt=full_report&list_uids=510342), [TMBIM4](http://www.ncbi.nlm.nih.gov/entrez/query.fcgi?db=gene&cmd=retrieve&dopt=full_report&list_uids=513242), [LLPH](http://www.ncbi.nlm.nih.gov/entrez/query.fcgi?db=gene&cmd=retrieve&dopt=full_report&list_uids=510518) |  |

**S1 Table. Chromosome (Chr), location, identification of genes and proportion of variance (VAR) explained by windows with largest effects on marbling^a^.**

^a^NCBI Symbol (Assembly UMD3.1, annotation release 103).
