# Supplementary material for: Genome-Wide Association Study of Meat Quality Traits in Nellore Cattle
Source: PLoS One. 2016 Jun 30;11(6):e0157845. doi: 10.1371/journal.pone.0157845 (PMC4928802; doi:10.1371/journal.pone.0157845)
Supplement: S2 Table — aNCBI Symbol (Assembly UMD3.1, annotation release 103). (DOCX) [file pone.0157845.s003.docx]

**S2 Table. Chromosome (Chr), location, identification of genes and proportion of**

**variance (VAR) explained by windows with largest effects on tenderness^a^.**

| Chr | Location (bp) | Genes | VAR (%) |
| --- | --- | --- | --- |
| 8 | 76050318-76880736 | [APTX](http://www.ncbi.nlm.nih.gov/entrez/query.fcgi?db=gene&cmd=retrieve&dopt=full_report&list_uids=359714), [LOC104969407](http://www.ncbi.nlm.nih.gov/entrez/query.fcgi?db=gene&cmd=retrieve&dopt=full_report&list_uids=104969407), [DNAJA1](http://www.ncbi.nlm.nih.gov/entrez/query.fcgi?db=gene&cmd=retrieve&dopt=full_report&list_uids=528862), [SMU1](http://www.ncbi.nlm.nih.gov/entrez/query.fcgi?db=gene&cmd=retrieve&dopt=full_report&list_uids=540842) | 0.44448 |
|  |  | [LOC100848199](http://www.ncbi.nlm.nih.gov/entrez/query.fcgi?db=gene&cmd=retrieve&dopt=full_report&list_uids=100848199), [B4GALT1](http://www.ncbi.nlm.nih.gov/entrez/query.fcgi?db=gene&cmd=retrieve&dopt=full_report&list_uids=281781), [SPINK4](http://www.ncbi.nlm.nih.gov/entrez/query.fcgi?db=gene&cmd=retrieve&dopt=full_report&list_uids=616822), [NOL6](http://www.ncbi.nlm.nih.gov/entrez/query.fcgi?db=gene&cmd=retrieve&dopt=full_report&list_uids=782674) |  |
|  |  | [BAG1](http://www.ncbi.nlm.nih.gov/entrez/query.fcgi?db=gene&cmd=retrieve&dopt=full_report&list_uids=613855), [CHMP5](http://www.ncbi.nlm.nih.gov/entrez/query.fcgi?db=gene&cmd=retrieve&dopt=full_report&list_uids=539781), [NFX1](http://www.ncbi.nlm.nih.gov/entrez/query.fcgi?db=gene&cmd=retrieve&dopt=full_report&list_uids=515680), [AQP7](http://www.ncbi.nlm.nih.gov/entrez/query.fcgi?db=gene&cmd=retrieve&dopt=full_report&list_uids=615498), [AQP3](http://www.ncbi.nlm.nih.gov/entrez/query.fcgi?db=gene&cmd=retrieve&dopt=full_report&list_uids=780866), [UBAP2](http://www.ncbi.nlm.nih.gov/entrez/query.fcgi?db=gene&cmd=retrieve&dopt=full_report&list_uids=506577) |  |
|  |  | [UBE2R2](http://www.ncbi.nlm.nih.gov/entrez/query.fcgi?db=gene&cmd=retrieve&dopt=full_report&list_uids=537885), [DCAF12](http://www.ncbi.nlm.nih.gov/entrez/query.fcgi?db=gene&cmd=retrieve&dopt=full_report&list_uids=525161) |  |
| 7 | 83027620-83594528 | [DHFR](http://www.ncbi.nlm.nih.gov/entrez/query.fcgi?db=gene&cmd=retrieve&dopt=full_report&list_uids=508809), [MSH3](http://www.ncbi.nlm.nih.gov/entrez/query.fcgi?db=gene&cmd=retrieve&dopt=full_report&list_uids=616744), [RASGRF2](http://www.ncbi.nlm.nih.gov/entrez/query.fcgi?db=gene&cmd=retrieve&dopt=full_report&list_uids=524957), [CKMT2](http://www.ncbi.nlm.nih.gov/entrez/query.fcgi?db=gene&cmd=retrieve&dopt=full_report&list_uids=538944) | 0.40279 |
| 5 | 77482400-78409153 | [YARS2](http://www.ncbi.nlm.nih.gov/entrez/query.fcgi?db=gene&cmd=retrieve&dopt=full_report&list_uids=536956), [DNM1L](http://www.ncbi.nlm.nih.gov/entrez/query.fcgi?db=gene&cmd=retrieve&dopt=full_report&list_uids=540892), [LOC101907810](http://www.ncbi.nlm.nih.gov/entrez/query.fcgi?db=gene&cmd=retrieve&dopt=full_report&list_uids=101907810), [FGD4](http://www.ncbi.nlm.nih.gov/entrez/query.fcgi?db=gene&cmd=retrieve&dopt=full_report&list_uids=505234), | 0.37767 |
|  |  | BICD1, [LOC782092](http://www.ncbi.nlm.nih.gov/entrez/query.fcgi?db=gene&cmd=retrieve&dopt=full_report&list_uids=782092), [KIAA1551](http://www.ncbi.nlm.nih.gov/entrez/query.fcgi?db=gene&cmd=retrieve&dopt=full_report&list_uids=510651) |  |
| 14 | 53788297-54583246 | [LOC101905320](http://www.ncbi.nlm.nih.gov/entrez/query.fcgi?db=gene&cmd=retrieve&dopt=full_report&list_uids=101905320) | 0.37693 |

^a^NCBI Symbol (Assembly UMD3.1, annotation release 103).
